# Levels of Inflammatory Cytokines IL-1*β*, IL-6, IL-8, IL-17A, and TNF-*α* in Aqueous Humour of Patients with Diabetic Retinopathy

**DOI:** 10.1155/2018/8546423

**Published:** 2018-04-04

**Authors:** Songfu Feng, Honghua Yu, Ying Yu, Yu Geng, Dongli Li, Chun Yang, Qingjun Lv, Li Lu, Ting Liu, Guodong Li, Ling Yuan

**Affiliations:** ^1^Department of Ophthalmology, ZhuJiang Hospital of Southern Medical University, Guangzhou 510280, China; ^2^Department of Ophthalmology, Guangdong Eye Institute, Guangdong General Hospital, Guangdong Academy of Medical Sciences, Guangzhou 510080, China; ^3^Department of Ophthalmology, The First Affiliated Hospital of Kunming Medical College, Kunming 650031, China; ^4^Gejiu People's Hospital, Gejiu 661000, China; ^5^The Second People's Hospital of Jiangxi, Nanchang 330000, China

## Abstract

Diabetic retinopathy is the leading cause of blindness in working age individuals in developed countries. However, the role of inflammation in the pathogenesis of DR is not completely understood. This is an observational clinical research enrolling 80 type II diabetic patients who had undergone cataract surgeries either with DR or without DR. All cases were further categorized by the proliferative stages of retinal neovascularization and by the lengths of diabetic history. The levels of inflammatory cytokines including IL-1*β*, IL-6, IL-8, IL-17, and TNF-*α* in aqueous humour were tested. Results in this study indicated that these cytokine levels were increased in DR patients and might have a synergistic effect on the pathogenesis of this disease. They were also elevated along with the progression of neovascularization, reflecting the severity of DR. The results also suggested that for diabetic patients, the higher these levels are, the sooner retinal complications might appear. In conclusion, the levels of inflammatory cytokines IL-1*β*, IL-6, IL-8, IL-17A, and TNF-*α* in aqueous humour may be associated with the pathogenesis, severity, and prognosis of DR.

## 1. Introduction

Diabetic retinopathy (DR), the most prevalent microvascular complication of diabetes mellitus (DM), is also the leading cause of blindness in working age individuals in developed countries [1]. Compared to nondiabetic subjects, diabetic patients present a significant 25-fold increase in blindness [2].

According to the proliferative status of retinal neovascularization, DR could be presented in two stages as nonproliferative stage (NPDR) and proliferative or neovascular stage (PDR) [3]. The pathological manifestations of DR include decreased retinal perfusion, thickened endothelial basement membrane in capillaries, loss of pericytes, increase in vasopermeability, and retinal neovascularization [4–6]. As a result, macular edema, vitreous hemorrhage, and tractional detachment of the retina are most responsible for vision loss [7].

The pathogenesis of DR has been described as the synergistic reaction of multiple factors, involving hyperglycemia, hemodynamic disorder, oxidative stress, mitochondrial dysfunction, and so on [8–12]. In recent years, a new argument has emerged that inflammation is an important factor in the occurrence and development of DR [13–20]. The first report indicating that inflammation contributes to the development of DR was from Powell and Field's study in 1964 showing that diabetic patients who had taken salicylates to treat rheumatoid arthritis had an incidence of DR lower than expected [21]. After that, more evidences were revealed to connect DR with inflammation [22–25]. However, the role of inflammation in the pathogenesis of DR is not completely understood and needs further exploration [3], especially concerning inflammatory cytokines, the biological markers of inflammatory response.

The aim of the present study was to compare the levels of inflammatory cytokines including interleukin- (IL-) 1*β*, IL-6, IL-8, IL-17, and tumor necrosis factor-*α* (TNF-*α*) in aqueous humour of patients either with DR or without DR, as well as to compare them in different proliferative stages of retinal neovascularization and in different lengths of diabetic history, in order to further explore the role of inflammatory cytokines in DR development.

## 2. Materials and Methods

The present study was approved by the Clinical Research Ethics Committee of the First Affiliated Hospital of Kunming Medical University and followed the tenets of the Declaration of Helsinki. Informed consents were acquired from all participants before experiment.

### 2.1. Study Population

This is a nonrandomized single-center observational clinical research enrolling 80 diabetic patients who were hospitalized in the First Affiliated Hospital of Kunming Medical University (Yunnan province, China) for cataract surgeries, from January 2015 to January 2016. Exclusion xcriteria are the following: (1) patients treated with anticoagulants; (2) patients with acute metabolic disorders, for example, ketoacidosis, or hyperosmolar syndrome; (3) patients with diabetic macrovascular complications; (4) patients with a history of cardiocerebrovascular disease; (5) patients with abnormal liver or kidney function; (6) patients with hypertension; (7) patients with mental illness; (8) patients with type I diabetes; and (9) patients who had received laser, anti-VEGF therapy, or triamcinolone treatment in the prior year.

### 2.2. Case Categorization

Among all 80 patients with DM, 40 of them were diagnosed with DR (categorized into the DR group), while the rest without DR (categorized into the DM group). All cases were further categorized according to the lengths of their diabetic history, as the 5-year DR group, 10-year DR group and the 5-year DM group, 10-year DM group. The DR group was further categorized according to the proliferative stages of retinal neovascularization, as the 5-year nonproliferative DR group (5-year NPDR group), 5-year proliferative DR group (5-year PDR group), and the 10-year NPDR group, 10-year PDR group.

### 2.3. General Data Acquisition

General data of each patient was collected including age, gender, height, weight, and medical history (e.g. diabetic history and medication history). Body mass index (BMI) is a common index of obesity and calculated by weight and height squared (kg/m^2^) [26]. Data from patient records/information were anonymized and deidentified before analysis.

### 2.4. Ophthalmologic Examination

Visual acuity and intraocular pressure of each patient were measured, respectively, through Snellen charts and Goldmann applanation tonometer. All cases were examined by slit-lamp microscopic examination, fundus colorized photography (Carl Zeiss, Germany), fundus fluorescein angiography (FFA), and optical coherence tomography (Carl Zeiss, Germany). During FFA, 5 ml 10% sodium fluorescein was injected into the antecubital vein and digital images were taken. Retinopathy was classified into nonproliferative DR and proliferative DR by FFA results according to the Early Treatment of Diabetic Retinopathy Study (ETDRS) [27].

### 2.5. Sample Assay

0.2 ml aqueous humour sample was drawn out of each patient during cataract surgery and preserved in −80°C refrigerator for later assays. Then, levels of inflammatory cytokines including IL-1*β*, IL-6, IL-8, IL-17, and TNF-*α* in aqueous humour were assayed by enzyme-linked immunosorbent assay (ELISA). 6 ml blood sample was drawn out of each patient though median cubital vein, respectively, in the morning with empty stomach and at two-hour postprandial moment. Glycosylated hemoglobin A1c (HbA1c), fasting blood glucose (FBG), and 2 h postprandial blood glucose (2hPG) were assayed by automatic biochemical analyzer.

### 2.6. Data Analysis Methods

Data were analyzed using SPSS 20.0 statistics software and evaluated by means and standard deviations ($\bar{x}\pm s$). *t*-test was used to compare data between groups. Pearson correlation analysis was adopted to analyze the relations among the inflammatory cytokines. Multiple stepwise regression analysis was applied to detect the significance of influential factors. *p* < 0.05 was considered statistically significant.

## 3. Results

### 3.1. General Information

A total of 80 patients, including 36 males and 44 females, were enrolled in the present study. There were each 20 cases in the 5-year DR group, 10-year DR group and 5-year DM group, 10-year DM group, with no statistical differences of the construction of age, gender, or BMI among the groups (all *p* > 0.05) (Table 1).

### 3.2. Biochemical Assay Results of Peripheral Blood

FBG, 2hPG, and HbA1c in peripheral blood samples were assayed. There were no statistical differences of these assay results among the groups (all *p* > 0.05) (Table 1).

### 3.3. Inflammatory Cytokine Levels in Aqueous Humour

**Figure 1:**
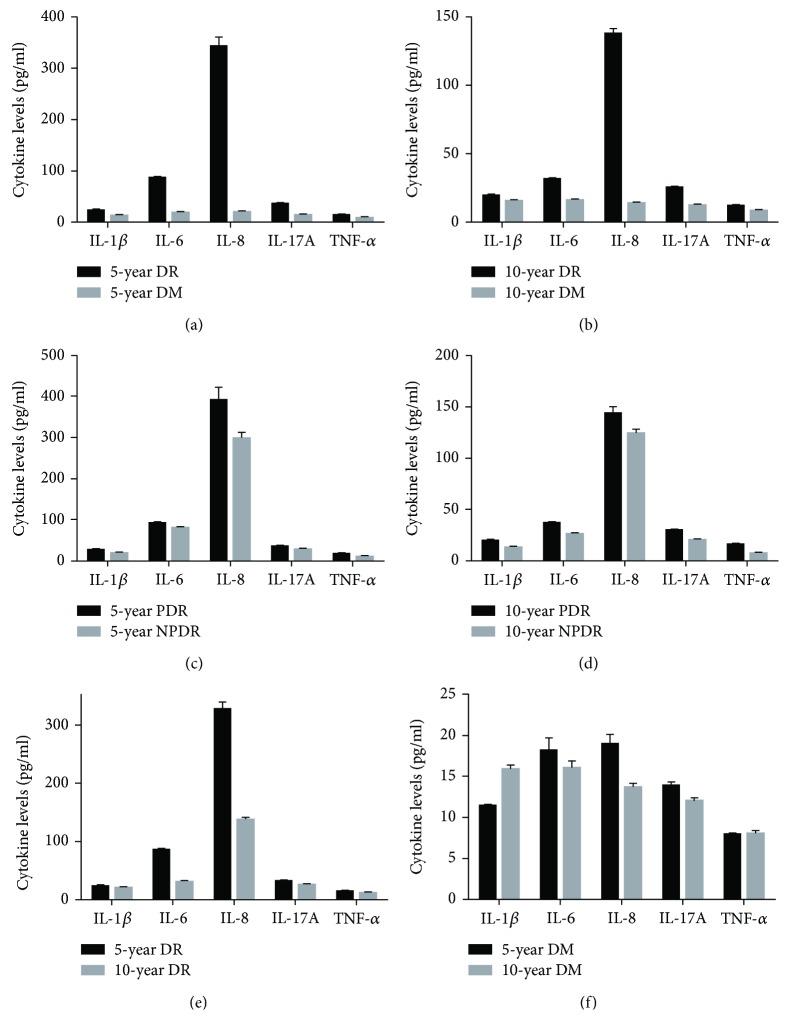
Histogram of inflammatory cytokine levels in aqueous humour. (a) Histogram of each cytokine in the 5-year DR group and 5-year DM group. All tested cytokines were higher in the 5-year DR group (all *p* < 0.05). (b) Histogram of each cytokine in the 10-year DR group and 10-year DM group. All tested cytokines were higher in the 10-year DR group (all *p* < 0.05). (c) Histogram of each cytokine in the 5-year PDR group and 5-year NPDR group. All tested cytokines were higher in the 5-year PDR group (all *p* < 0.05). (d) Histogram of each cytokine in the 10-year PDR group and 10-year NPDR group. All tested cytokines were higher in the 10-year PDR group (all *p* < 0.05). (e) Histogram of each cytokine in the 5-year DR group and 10-year DR group. All tested cytokines were higher in the 5-year DR group (all *p* < 0.05). (f) Histogram of each cytokine in the 5-year DM group and 10-year DM group. There were no statistical differences in all tested cytokines in these two groups (all *p* > 0.05).

In aqueous humour samples, the inflammatory cytokines including IL-1*β*, IL-6, IL-8, IL-17A, and TNF-*α* were assayed. The average levels of these cytokines in the 5-year DR group were all significantly higher than those in the 5-year DM group (all *p* < 0.05) (Table 2, Figure 1(a)). And the average levels of these cytokines in the 10-year DR group were also all significantly higher than those in the 10-year DM group (all *p* < 0.05) (Table 2, Figure 1(b)).

Regarding different stages of proliferative retinal neovascularization, the 5-year DR group consisted of 11 cases in the 5-year NPDR group and 9 cases in the 5-year PDR group, while the 10-year DR group consisted of 10 cases in the 10-year NPDR group and 10 cases in the 10-year PDR group. The average levels of cytokines IL-1*β*, IL-6, IL-8, IL-17A, and TNF-*α* in the 5-year PDR group were all significantly higher than those in the 5-year NPDR group (all *p* < 0.05) (Table 2, Figure 1(c)). And the average levels of these cytokines in the 10-year PDR group were also all significantly higher than those in the 10-year NPDR group (all *p* < 0.05) (Table 2, Figure 1(d)).

With respect to different lengths of diabetic history, the average levels of these cytokines in the 5-year DR group were all significantly higher than those in the 10-year DR group (*t* = 15.134, 28.596, 16.068, 13.978, and 7.768, resp.; all *p* = 0.001) (Figure 1(e)). Whereas, there were no statistical differences of average levels of these cytokines between the 5-year DM group and 10-year DM group (*t* = −8.518, 1.175, 4.755, 3.648, −0.331; *p* = 0.120, 0.254, 0.360, 0.482, and 0.744, resp.) (Figure 1(f)).

In addition, between each two of these cytokines, there was a positive correlation in the 5-year DR group and in 10-year DR group (all *p* < 0.05). Nevertheless, there was no such correlation in the 5-year DM group or in 10-year DM group (all *p* > 0.05) (see supplementary material available here).

## 4. Discussion

In 2014, 387 million people (8.3% of the population worldwide) had diabetes, a chronic metabolic disease with increasing incidence and prevalence. It is estimated that within 20 years, this number would increase to 592 million [28]. Vascular complications of diabetes could be categorized into macrovascular complications and microvascular complications. And the most common microvascular complication is DR. According to a multi-hospital-based cross-sectional study in 2017, the age-gender-standardised prevalence of DR in China was 27.9%, similar to that in other studies from Western countries and other Asian countries [29]. DR is also a major cause of blindness, as patients could lose sight due to diabetic macular edema (DME) and/or PDR during the development of DR [30]. Therefore, to understand the pathogenesis of DR is with great significance.

In recent years, evidences have emerged showing that chronic low-grade inflammation of retina is important to the pathogenesis of DR, as it contributes to the development of edema and neovascularization [3, 14, 25, 31, 32]. During the development of DR, the underlying mechanisms have been described as the following four biochemical pathways: increase in advanced glycation end products (AGEs) formation, increase in polyol pathway flux, increase in hexosamine pathway flux, and activation of protein kinase C (PKC) isoforms [33]. Each pathway could also contribute to the upregulation of inflammatory cytokines [20]. But how exactly do these cytokines manifest in the aqueous humour of DR patients is not clear.

Thus, the present study was designed to compare the manifestations of inflammatory cytokines including IL-1*β*, IL-6, IL-8, IL-17, and TNF-*α* in aqueous humour of patients either with DR or without DR. This observational clinical study enrolled 80 diabetic patients who had undergone cataract surgeries. Half the number of them have DR and half the number without. And in each of the above two groups, there were also half the number of patients with 5 years of diabetic history and half the number of patients with 10 years of diabetic history. Neither did age, gender, BMI, nor peripheral blood biochemical assays results, including FBG, 2hPG, and HbA1c, showed any statistical differences among the groups, which in certain degree ruled out some factors that could bias the cytokine levels. Furthermore, none of the cases in our study had received laser, anti-VEGF therapy, or triamcinolone treatment in the last one year. In this way, we could possibly rule out the effect of these treatments on the level of interleukin in the aqueous humour [34, 35].

Results in our study showed that in aqueous humour, the average levels of inflammatory cytokines IL-1*β*, IL-6, IL-8, IL-17A, and TNF-*α* in the 5-year DR group and 10-year DR group were higher than those in the 5-year DM group and 10-year DM group, respectively (all *p* < 0.05). In addition, between each two of these cytokines, there was a positive correlation in the 5-year DR group and in 10-year DR group (all *p* < 0.05), while there was no such correlation in the 5-year DM group or 10-year DM group (all *p* > 0.05). These results indicated that cytokines IL-1*β*, IL-6, IL-8, IL-17A, and TNF-*α* in aqueous humour were increased in DR patients and might have a synergistic effect on the pathogenesis of this disease.

Results in our study also demonstrated that in aqueous humour, the average levels of IL-1*β*, IL-6, IL-8, IL-17A, and TNF-*α* in the 5-year PDR group and 10-year PDR group were higher than those in the 5-year NPDR group and 10-year NPDR group, respectively (all *p* < 0.05). Therefore, we inferred that, in the aqueous humour of DR patients, IL-1*β*, IL-6, IL-8, IL-17A, and TNF-*α* were elevated to the certain degree according to the progression of pathological neovascularization and that monitoring these cytokines dynamically could aid us in determining the severity of proliferation, which also reflects the severity of DR.

Furthermore, results in our study manifested that in aqueous humour, the average levels of IL-1*β*, IL-6, IL-8, IL-17A, and TNF-*α* in the 5-year PDR group were higher than those in the 10-year PDR group (all *p* < 0.05). The reason why the 10-year data is better than those at 5 years might be that if DR was diagnosed on the 5th year of DM, it might suggest that the patient had not been under an effective glycemic control. Otherwise, the pathogenesis process of DR could be slower, with the diagnosis of DR being delayed. That is why patients diagnosed of DR on the 10th year of DM were likely to have a better data than those who were diagnosed of DR on the 5th year of DM. Also, with effective glycemic control, the glycemic level could be reduced over time, which is in accordance with a study led by Holman et al. [36]. This manifestation implied that for diabetic patients, the higher these cytokine levels are, the sooner retinal complications might appear.

## 5. Conclusions

The results of the present study suggested that the levels of inflammatory cytokines IL-1*β*, IL-6, IL-8, IL-17A, and TNF-*α* in aqueous humour may be associated to the pathogenesis, severity, and prognosis of DR. Studying the inflammatory manifestations in DR patients could provide great values for developing new strategies of diagnosis and treatment. More studies are needed to concentrate on treating DR by reducing these inflammatory factors.

## Figures and Tables

**Table 1:** General information and biochemical assay results of each group.

Groups	Case number	Age ($\bar{x}\pm s$)	Gender^∗^	BMI^∗^ ($\bar{x}\pm s$)	FBG^∗^ (mmol/l)	2hPG^∗^ (mmol/l)	HbA1c^∗^ (%)
5-year DR	20	58.5 ± 8.9	8/12	23.4 ± 1.0	8.7 ± 0.5	12.3 ± 3.4	11.2 ± 2.6
10-year DR	20	59.3 ± 7.2	9/11	23.7 ± 0.9	8.5 ± 0.8	11.9 ± 3.6	10.4 ± 3.1
5-year DM	20	58.4 ± 5.3	10/10	22.9 ± 0.8	8.5 ± 0.9	12.0 ± 2.8	10.9 ± 3.5
10-year DM	20	58.6 ± 7.5	9/11	23.4 ± 0.6	8.8 ± 0.3	12.1 ± 2.7	11.0 ± 2.8
*p* value		>0.05	>0.05	>0.05	>0.05	>0.05	>0.05

^∗^Gender: male/female; ^∗^BMI: body mass index; ^∗^FBG: fasting blood glucose; ^∗^2hPG: 2 hour postprandial blood glucose; ^∗^HbA1c: glycosylated hemoglobin A1c.

**Table 2:** Average levels of each inflammatory cytokine in aqueous humour ($\bar{x}\pm s$).

Groups	Case number	IL-1*β* ^∗^ (pg/ml)	IL-6 (pg/ml)	IL-8 (pg/ml)	IL-17A (pg/ml)	TNF-*α* ^∗^ (pg/ml)
5-year DR group	20	22.109 ± 0.34	86.324 ± 1.54	342.782 ± 18.55	32.751 ± 0.68	14.065 ± 0.23
5-year DM group	20	11.415 ± 0.25	18.221 ± 1.45	19.009 ± 1.13	13.875 ± 0.42	7.944 ± 0.22
*t* value		15.43	39.01	27.78	21.42	19.15
*p* value		0.001	0.001	0.001	0.002	0.001
10-year DR group	20	20.589 ± 0.24	31.480 ± 1.35	137.903 ± 3.90	25.460 ± 0.59	11.765 ± 0.18
10-year DM group	20	15.845 ± 0.53	16.080 ± 0.79	13.662 ± 0.53	12.084 ± 0.45	8.056 ± 0.29
*t* value		1.011	10.477	30.598	17.670	11.499
*p* value		0.005	0.001	0.003	0.001	0.001
5-year PDR group	9	24.127 ± 0.59	91.434 ± 2.12	384.171 ± 30.83	35.784 ± 0.91	16.432 ± 0.24
5-year NPDR group	11	18.229 ± 0.50	81.838 ± 2.33	300.930 ± 12.46	31.912 ± 0.96	12.826 ± 0.21
*t* value		4.958	1.887	2.444	2.532	13.197
*p* value		0.001	0.002	0.001	0.001	0.001
10-year PDR group	10	22.667 ± 2.27	35.987 ± 0.17	144.437 ± 5.77	30.352 ± 0.90	12.855 ± 0.20
10-year NPDR group	10	18.293 ± 1.50	27.190 ± 0.37	124.369 ± 1.05	20.568 ± 0.90	9.670 ± 0.17
*t* value		3.137	19.392	3.375	16.707	67.498
*p* value		0.002	0.001	0.001	0.001	0.001

All *p* values shown in this table are less than 0.05; ^∗^IL: interleukin; ^∗^TNF: tumor necrosis factor.

## Supplementary Materials

Supplementary Materials Supplementary Table: the correlation analysis results of each two of the assayed cytokines.

## References

[1] Thylefors B., Négrel A. D., Pararajasegaram R., Dadzie K. Y. (1995). Global data on blindness. *Bulletin of the World Health Organization*.

[2] Ling R., Ramsewak V., Taylor D., Jacob J. (2002). Longitudinal study of a cohort of people with diabetes screened by the Exeter Diabetic Retinopathy Screening Programme. *Eye*.

[3] Tang J., Kern T. S. (2011). Inflammation in diabetic retinopathy. *Progress in Retinal and Eye Research*.

[4] Alder V. A., Su E. N., Yu D. Y., Cringle S. J., Yu P. K. (1997). Diabetic retinopathy: early functional changes. *Clinical and Experimental Pharmacology and Physiology*.

[5] Barot M., Gokulgandhi M. R., Patel S., Mitra A. K. (2013). Microvascular complications and diabetic retinopathy: recent advances and future implications. *Future Medicinal Chemistry*.

[6] Bhagat N., Grigorian R. A., Tutela A., Zarbin M. A. (2009). Diabetic macular edema: pathogenesis and treatment. *Survey of Ophthalmology*.

[7] Basu S., Zethelius B., Helmersson J., Berne C., Larsson A., Arnlöv J. (2011). Cytokine-mediated inflammation is independently associated with insulin sensitivity measured by the euglycemic insulin clamp in a community-based cohort of elderly men. *International Journal of Clinical and Experimental Medicine*.

[8] Klein R. (1995). Hyperglycemia and microvascular and macrovascular disease in diabetes. *Diabetes Care*.

[9] Kanwar M., Chan P. S., Kern T. S., Kowluru R. A. (2007). Oxidative damage in the retinal mitochondria of diabetic mice: possible protection by superoxide dismutase. *Investigative Ophthalmology & Visual Science*.

[10] Kowluru R. A., Tang J., Kern T. S. (2001). Abnormalities of retinal metabolism in diabetes and experimental galactosemia. VII. Effect of long-term administration of antioxidants on the development of retinopathy. *Diabetes*.

[11] Madsen-Bouterse S. A., Zhong Q., Mohammad G., Ho Y. S., Kowluru R. A. (2010). Oxidative damage of mitochondrial DNA in diabetes and its protection by manganese superoxide dismutase. *Free Radical Research*.

[12] Scarpulla R. C. (2012). Nucleus-encoded regulators of mitochondrial function: integration of respiratory chain expression, nutrient sensing and metabolic stress. *Biochimica et Biophysica Acta (BBA) - Gene Regulatory Mechanisms*.

[13] Sahakyan K., Klein B. E. K., Lee K. E., Tsai M. Y., Klein R. (2010). Inflammatory and endothelial dysfunction markers and proteinuria in persons with type 1 diabetes mellitus. *European Journal of Endocrinology*.

[14] Adamis A. P. (2002). Is diabetic retinopathy an inflammatory disease?. *The British Journal of Ophthalmology*.

[15] Gologorsky D., Thanos A., Vavvas D. (2012). Therapeutic interventions against inflammatory and angiogenic mediators in proliferative diabetic retinopathy. *Mediators of Inflammation*.

[16] Kaštelan S., Tomić M., Gverović Antunica A., Salopek Rabatić J., Ljubić S. (2013). Inflammation and pharmacological treatment in diabetic retinopathy. *Mediators of Inflammation*.

[17] Perez V. L., Caspi R. R. (2015). Immune mechanisms in inflammatory and degenerative eye disease. *Trends in Immunology*.

[18] Rangasamy S., McGuire P. G., Franco Nitta C., Monickaraj F., Oruganti S. R., Das A. (2014). Chemokine mediated monocyte trafficking into the retina: role of inflammation in alteration of the blood-retinal barrier in diabetic retinopathy. *PLoS One*.

[19] Sasongko M. B., Wong T. Y., Jenkins A. J., Nguyen T. T., Shaw J. E., Wang J. J. (2015). Circulating markers of inflammation and endothelial function, and their relationship to diabetic retinopathy. *Diabetic Medicine*.

[20] Semeraro F., Cancarini A., dell’Omo R., Rezzola S., Romano M. R., Costagliola C. (2015). Diabetic retinopathy: vascular and inflammatory disease. *Journal of Diabetes Research*.

[21] Powell E. D., Field R. A. (1964). Diabetic retinopathy and rheumatoid arthritis. *The Lancet*.

[22] Brucklacher R. M., Patel K. M., VanGuilder H. D. (2008). Whole genome assessment of the retinal response to diabetes reveals a progressive neurovascular inflammatory response. *BMC Medical Genomics*.

[23] Adamis A. P., Berman A. J. (2008). Immunological mechanisms in the pathogenesis of diabetic retinopathy. *Seminars in Immunopathology*.

[24] Kaul K., Hodgkinson A., M. Tarr J., M. Kohner E., Chibber R. (2010). Is inflammation a common retinal-renal-nerve pathogenic link in diabetes?. *Current Diabetes Reviews*.

[25] Kern T. S. (2007). Contributions of inflammatory processes to the development of the early stages of diabetic retinopathy. *Experimental Diabetes Research*.

[26] Kumanyika S. K., Obarzanek E., Stettler N. (2008). Population-based prevention of obesity: the need for comprehensive promotion of healthful eating, physical activity, and energy balance: a scientific statement from American Heart Association Council on Epidemiology and Prevention, Interdisciplinary Committee for Prevention (formerly the expert panel on population and prevention science). *Circulation*.

[27] Early Treatment Diabetic Retinopathy Study Research Group (1991). Grading diabetic retinopathy from stereoscopic color fundus photographs—an extension of the modified Airlie House classification. ETDRS report number 10. *Ophthalmology*.

[28] Guariguata L., Whiting D. R., Hambleton I., Beagley J., Linnenkamp U., Shaw J. E. (2014). Global estimates of diabetes prevalence for 2013 and projections for 2035. *Diabetes Research and Clinical Practice*.

[29] Zhang G., Chen H., Chen W., Zhang M. (2017). Prevalence and risk factors for diabetic retinopathy in China: a multi-hospital-based cross-sectional study. *The British Journal of Ophthalmology*.

[30] Roy S., Kern T. S., Song B., Stuebe C. (2017). Mechanistic insights into pathological changes in the diabetic retina: implications for targeting diabetic retinopathy. *The American Journal of Pathology*.

[31] Noda K., Nakao S., Ishida S., Ishibashi T. (2012). Leukocyte adhesion molecules in diabetic retinopathy. *Journal of Ophthalmology*.

[32] Rangasamy S., McGuire P. G., Das A. (2012). Diabetic retinopathy and inflammation: novel therapeutic targets. *Middle East African Journal of Ophthalmology*.

[33] Brownlee M. (2001). Biochemistry and molecular cell biology of diabetic complications. *Nature*.

[34] Shah A. R., Del Priore L. V. (2009). Duration of action of intravitreal ranibizumab and bevacizumab in exudative AMD eyes based on macular volume measurements. *British Journal of Ophthalmology*.

[35] Jonas J. B., Degenring R. F., Kamppeter B. A., Kreissig I., Akkoyun I. (2004). Duration of the effect of intravitreal triamcinolone acetonide as treatment for diffuse diabetic macular edema. *American Journal of Ophthalmology*.

[36] Holman R. R., Paul S. K., Bethel M. A., Matthews D. R., Neil H. A. W. (2008). 10-year follow-up of intensive glucose control in type 2 diabetes. *New England Journal of Medicine*.

