# Supplementary material for: Levels of Inflammatory Cytokines IL-1β, IL-6, IL-8, IL-17A, and TNF-α in Aqueous Humour of Patients with Diabetic Retinopathy
Source: J Diabetes Res. 2018 Apr 4;2018:8546423. doi: 10.1155/2018/8546423 (PMC5904804; doi:10.1155/2018/8546423)
Supplement: Supplementary Materials — Supplementary Table: the correlation analysis results of each two of the assayed cytokines. [file 8546423.f1.pdf]

Supplementary Table 1

The correlation analysis results of each two of the assayed cytokines

		IL*-6		IL-8		IL-17A		TNF*-α	
		r*	p*	r	p	r	p	r	p
5-year DR group	IL-1β	0.043	0.018**	0.123	0.014**	0.119	0.018**	0.211	0.030**
	IL-6			0.098	0.026**	0.375	0.004**	0.260	0.028**
	IL-8					0.463	0.012**	0.240	0.010**
	IL-17A							0.358	0.012**
10-year DR group	IL-1β	0.538	0.015**	0.166	0.009**	0.393	0.005**	0.190	0.024**
	IL-6			0.026	0.002**	0.228	0.001**	0.081	0.000**
	IL-8					0.122	0.027**	0.690	0.020**
	IL-17A							0.076	0.000**
5-year DM group	IL-1β	-0.042	0.859	-0.177	0.456	0.034	0.886	-0.802	0.737
	IL-6			0.261	0.267	-0.054	0.820	0.008	0.972
	IL-8					-0.052	0.829	0.137	0.564
	IL-17A							-0.355	0.126
10-year DM group	IL-1β	0.128	0.592	0.094	0.695	0.170	0.473	-0.207	0.381
	IL-6			-0.274	0.242	-0.140	0.557	0.130	0.586
	IL-8					0.057	0.810	-0.065	0.786
	IL-17A							-0.294	0.289

*IL: Interleukin.

*TNF: Tumor necrosis factor.

*r: The coefficients of correlation.

*p: $p<0.05$ was considered significant.

**$p<0.05$
